# Pathways for the precise prevention and improvement of mental health among university freshmen: a network analysis and simulated intervention study based on the biopsychosocial model

**DOI:** 10.3389/fpsyg.2026.1777211

**Published:** 2026-07-03

**Authors:** Wenjie Zhang, Yujing Xie, Lijun Ma

**Affiliations:** School of Public Health and Management, Guangzhou University of Chinese Medicine, Guangzhou, Guangdong, China

**Keywords:** biopsychosocial model, mental health of university freshmen, network analysis, psychological resilience, simulated intervention, stress, traditional Chinese medicine constitution

## Abstract

**Objective:**

Given the high prevalence of mental health problems among university freshmen and the limited explanatory capacity of traditional unidimensional models, this study adopts a biopsychosocial (BPS) framework. Network analysis combined with simulation techniques based on the Node Identify via Recursive Graphs (NIRA) algorithm was applied to explore system-level interactions and potential targets within the network.

**Methods:**

A total of 3,116 first-year university students were recruited. The network comprised biological-related functional indicators (e.g., TCM constitution types such as Qi stagnation), psychological symptoms (depression, anxiety, suicide risk), psychological traits (resilience, emotion regulation, insight), and social factors (perceived stress, childhood trauma, and social support). An Ising network model was estimated, and centrality and bridge indices were calculated. Simulation analyses were conducted by manipulating node activation probabilities to examine potential changes in overall network activation.

**Results:**

Psychological resilience emerged as the central hub node, while perceived stress acted as the strongest bridge node linking social and psychological domains. Simulation analyses suggested that reductions in stress-related nodes and improvements in Qi stagnation–related indicators were associated with decreases in overall psychological network activation.

**Conclusion:**

These findings support the utility of a network-based BPS framework for understanding freshmen’s mental health. Psychological resilience and perceived stress may represent important components within the system. However, simulation findings should be interpreted cautiously, as they do not imply causal intervention effects.

## 1 Introduction

The global population of university students is facing increasingly severe mental health challenges, a trend consistently documented across diverse geographical and cultural contexts. A World Health Organization study involving 13,984 first-year students from 21 universities across eight countries reported that 31% screened positive for at least one mental disorder within the previous 12 months, highlighting the widespread and cross-national nature of psychological problems in this population (Auerbach et al., 2018).

In China, the mental health status of university students has shown a marked deterioration over the past decade, with substantial increases in anxiety, depression, sleep disturbances, and suicide-related behaviors. The Report on the Development of National Mental Health in China (2019–2020) indicated that 18.5% of college students exhibited depressive tendencies, 4.2% were at high risk for depression, and 8.4% showed anxiety tendencies. More recent data from the China National Mental Health Report (2022) further revealed increases in the detected risk rates of depression (21.48%) and anxiety (45.28%). Similarly, the 2022 National Blue Book on Depression reported that students accounted for approximately 50% of individuals diagnosed with depression (Zhang Mengmeng, 2024).

The transition into university represents a critical developmental period associated with elevated vulnerability to psychological maladjustment. First-year students are required to navigate multiple concurrent stressors, including role transitions, independent living, academic demands, interpersonal reorganization, and identity development (Credé and Niehorster, 2012). Individuals aged 18–25 are typically in the stage of emerging adulthood, during which neurodevelopmental processes such as prefrontal cortex maturation remain ongoing, potentially increasing sensitivity to environmental stressors (Arnett, 2004). Empirical evidence from China indicates that the detection rate of depression among first-year university students reaches 28.4% (Liu et al., 2016), while the prevalence of suicidal ideation is approximately 9% (Koushik et al., 2025). Collectively, these findings underscore the heightened psychological vulnerability of this population and the need for more refined analytical approaches.

Despite increasing recognition of the multifactorial nature of mental health problems, many existing studies continue to rely on linear, variable-centered, or unidimensional approaches. Although such approaches are useful for estimating the independent effects of predictors or testing latent constructs, they are less suited to examining how biological, psychological, and social factors may interact with and reinforce one another within a complex system. The biopsychosocial (BPS) model (Engel, 1977) posits that mental health outcomes arise from dynamic interactions among biological, psychological, and social systems (Papadimitriou, 2017; Borrell-Carrió et al., 2004). Empirical evidence supports this framework, indicating that biological-related functional factors such as Traditional Chinese Medicine (TCM) constitution types—particularly Qi stagnation constitution—are associated with psychological risk (Jinhe and Miao, 2024; Huang et al., 2022). At the psychological level, resilience has been consistently linked to more favorable mental health outcomes, whereas social stressors represent key environmental influences (Park et al., 2023). Although the BPS model provides a comprehensive conceptual framework, its empirical operationalization remains limited.

Network analysis is particularly relevant in this context because it enables the investigation of system-level relationships among observable components (Borsboom, 2017). Rather than focusing only on the isolated contribution of each factor, the network approach allows researchers to examine how components are conditionally connected, which nodes occupy structurally central positions, and which nodes may serve as bridges linking different domains (Cai et al., 2022; Ouyang et al., 2024). These features are directly aligned with the present study’s aims: to identify potentially influential biopsychosocial factors, to clarify cross-domain connections within the mental health system of first-year university students, and to explore whether these interaction patterns differ by gender. Recent studies applying network analysis to university student populations have identified key roles of depression, anxiety, and sleep-related symptoms in maintaining psychological distress (Sun et al., 2024; Sun et al., 2025). However, relatively few studies have incorporated biological-related indicators within a comprehensive BPS framework.

In addition, gender differences in mental health are well documented, with female students generally reporting higher levels of internalizing symptoms (Oliveira et al., 2025). However, the mechanisms underlying these differences remain unclear from a systems perspective. Accordingly, the present study further explores gender-specific network structures.

To address these gaps, the present study integrates biological-related functional indicators (TCM constitution), psychological symptoms and traits, and social environmental factors into a unified network framework. Furthermore, simulation analyses based on the Node Identify via Recursive Graphs (NIRA) algorithm were conducted to examine how changes in specific nodes are associated with variations in overall network activation (Robinaugh et al., 2020). It should be noted that these simulations represent model-based approximations rather than real-world interventions.

## 2 Methods

### 2.1 Participants

In September 2024, during freshman orientation week, 3,131 first-year university students were recruited for this study. The inclusion criteria were as follows: (1) full-time newly enrolled students, (2) aged 18 years or older, and (3) adequate Chinese reading comprehension ability. The studies involving humans were approved by the Second Affiliated Hospital of Guangzhou University of Chinese Medicine (IEC GL/07.0/01.1.). The studies were conducted in accordance with the Declaration of Helsinki. The participants provided their written informed consent to participate in this study.

To ensure data quality, a three-step validation procedure was implemented: (1) exclusion of participants younger than 18 years, (2) removal of questionnaires that failed embedded attention-check items, and (3) elimination of responses with abnormal completion times (i.e., less than 50% or greater than 200% of the median completion duration). Following data screening, 3,116 valid participants were retained, resulting in a retention rate of 99.5%. The final sample consisted of 1,252 males (40.2%) and 1,864 females (59.8%), with a mean age of 18.30 years (SD = 0.79).

### 2.2 Research instruments

The variables included in the network were selected according to the biopsychosocial framework of this study rather than solely on the basis of emotional distress. Specifically, the network comprised: (1) a biological-related functional indicator (TCM constitution), (2) psychological symptom indicators directly related to suicide risk, including anxiety, depression, suicidal ideation, and suicidal behavior, (3) psychological capacity and trait variables that may buffer or amplify symptom expression, including emotion regulation, resilience, and dispositional insight, and (4) social-contextual variables reflecting environmental adversity and support, including childhood trauma, perceived stress, and perceived social support.

#### 2.2.1 General information questionnaire

A self-developed demographic questionnaire was administered to collect information on participants’ age, ethnicity, academic year, affiliated college, major, class, and only-child status.

#### 2.2.2 Biological-related functional indicator

The constitution theory in Traditional Chinese Medicine (TCM) is grounded in the systematic classification of the abundance, depletion, deficiency, and excess of fundamental vital substances in the human body, including yin, yang, qi, blood, and body fluids. By integrating observable somatic traits, physiological manifestations (e.g., tongue appearance and pulse characteristics), and specific disease susceptibility tendencies, it establishes a comprehensive assessment framework that correlates internal physiological states with external life phenomena. TCM constitution does not represent a direct biological measurement comparable to physiological biomarkers or laboratory-based indicators. Rather, it reflects relatively stable patterns of self-perceived physical, psychological, and behavioral functioning conceptualized within Traditional Chinese Medicine. In the present study, TCM constitution was therefore included as a biological-related functional construct within an expanded biopsychosocial framework. This classification should be understood as a theoretical and functional categorization rather than evidence of objective biological status (Wang Qi et al., 2006).

In the present study, TCM constitution is conceptualized as a biological-related functional indicator rather than a direct biological measurement. It reflects integrated patterns of self-perceived physical, psychological, and behavioral functioning conceptualized within Traditional Chinese Medicine. Within the biopsychosocial framework, this construct is therefore treated as a system-level proxy of physiological susceptibility, while acknowledging its indirect and model-based nature.

Based on the foregoing, the Chinese Medicine Constitution Questionnaire (CCMQ-30) was employed as the research instrument for the biological-related functional indicators of this study.

The Chinese Medicine Constitution Questionnaire (CCMQ-30), developed by Zhu Yanbo et al. as a shortened version of the original 41-item instrument, was designed to assess constitution-based intervention effects while streamlining scale content (Yanbo et al., 2018). Item reduction was conducted through systematic psychometric procedures, including variability analysis, item–total correlation, Cronbach’s alpha analysis, factor analysis, critical ratio methods, and expert consultation.

The CCMQ-30 classifies individuals into nine constitution types: balanced, Qi-deficiency, Yang-deficiency, Yin-deficiency, phlegm-dampness, damp-heat, blood stasis, Qi stagnation, and inherited special constitution (Yanbo et al., 2018). In the present study, the CCMQ-30 demonstrated good internal consistency, with a Cronbach’s *α* of 0.798.

#### 2.2.3 Psychological dimension: symptom and trait assessment

##### 2.2.3.1 Psychological symptom assessment

Anxiety and depressive symptoms were assessed using standardized Beck inventories. Anxiety was measured with the Beck Anxiety Inventory (BAI), which is grounded in cognitive theory and conceptualizes anxiety as an affective–physiological–behavioral syndrome arising from negative cognitive schemas. The BAI consists of 21 items, and the Chinese version has demonstrated good reliability and normative validity; scores ≥16 (out of 63) indicate at least moderate anxiety (Beck et al., 1988; Jianrong et al., 2002). Depressive symptoms were assessed using the Beck Depression Inventory–II (BDI-II), which evaluates cognitive, affective, and somatic dimensions of depression. The 1996 revision of the BDI-II enhanced sensitivity to cognitive symptoms, with scores ≥19 (out of 63) indicating a moderate or higher risk of depression (Beck and Steer, 1996).

A dual-dimensional approach was adopted to separately assess suicidal ideation and suicidal behavior. Suicidal ideation was measured using the Self-rating Idea of Suicide Scale (SIOSS), developed by Xia Chaoyun for Chinese adolescents. The SIOSS comprises three clinical dimensions—hopelessness, optimism, and sleep—along with a concealment factor; positive suicidal ideation is defined by a total score ≥12 and a concealment score <4 (Jianhui, 2002). Suicidal behavior was assessed using the Suicidal Behaviors Questionnaire–Revised (SBQ-R), which captures lifetime suicidal behavior, recent intensity of ideation, disclosure of intent, and future likelihood across four items. Scores ≥7 (out of 18) indicate high suicide risk (Aloba et al., 2017). The combined use of these instruments enables comprehensive assessment of the suicide risk continuum from ideation to behavior. In the present study, all scales demonstrated good internal consistency, with Cronbach’s *α* coefficients ranging from 0.775 to 0.928.

##### 2.2.3.2 Assessment of psychological capacities and traits

Emotion regulation was assessed using the Difficulties in Emotion Regulation Scale (DERS), which evaluates adaptive emotional functioning based on a multidimensional framework. The DERS comprises 36 items covering six core dimensions, including emotional awareness, emotional clarity, acceptance of emotional responses, impulse control difficulties, difficulties in goal-directed behavior and limited access to effective emotion regulation strategies, with higher scores indicating greater difficulties in emotion regulation (Li et al., 2007).

Psychological resilience was measured using the Connor–Davidson Resilience Scale (CD-RISC). Normative studies among Chinese university students indicate that a total score ≥71 (out of 100) reflects a high level of resilience and serves as an important cutoff for identifying highly protected individuals (Connor and Davidson, 2003; Jianxin, 2005).

Dispositional insight was assessed using the Dispositional Insight Scale (DIS), which measures individuals’ tendency to experience sudden comprehension of problem essence during cognitive processing (i.e., “Aha!” experiences). This construct is considered a key mechanism underlying creative problem solving in neurocognitive research (Kounios and Beeman, 2014). The Chinese revised version employs a 7-point Likert scale across five items (1 = strongly disagree, 7 = strongly agree), yielding total scores ranging from 5 to 35.

In the present study, all scales demonstrated excellent internal consistency, with Cronbach’s *α* coefficients ranging from 0.893 to 0.973.

#### 2.2.4 Social dimension: assessment of stressors and support systems

Childhood experiences were assessed using the short form of the Childhood Trauma Questionnaire (CTQ-SF) (Thombs et al., 2007), which evaluates the severity of five categories of traumatic experiences, including emotional abuse and physical neglect. Cutoff scores for defining moderate-to-severe trauma were as follows: emotional abuse ≥13, physical abuse ≥10, sexual abuse ≥8, emotional neglect ≥15, and physical neglect ≥10.

Perceived stress was measured using the Perceived Stress Scale (PSS), which emphasizes the assessment of subjective stress experiences rather than objective stressors (Leung et al., 2010). The 14-item PSS comprises two orthogonal factors—perceived tension and perceived lack of control—with total scores ranging from 0 to 56. Based on established clinical grading criteria, total scores ≤28 indicate low stress, scores of 29–42 indicate moderate stress, and scores ≥43 indicate clinically significant stress; the scale has demonstrated good predictive validity in occupational populations.

Perceived social support was assessed using the Perceived Social Support Scale (PSSS) (Blumenthal et al., 1987), which captures the heterogeneity of social support sources by differentiating support networks into three dimensions: family support, peer support, and other support.

In the present study, all scales demonstrated good internal consistency, with Cronbach’s *α* coefficients ranging from 0.783 to 0.923.

The questionnaires described above served as measurement instruments from which the network variables were derived. The network variables were not represented by raw questionnaire scores. Instead, questionnaire items were first processed according to predefined criteria and subsequently aggregated into construct-level indicators, which were then transformed into binary variables for network estimation (van Borkulo et al., 2014). Therefore, all nodes entered into the network at the same construct level of analysis.

Consistent with the biopsychosocial framework and the network approach to psychopathology, the present study focused on theoretically meaningful constructs rather than on individual questionnaire dimensions or items. Accordingly, indicators were selected on the basis of the primary constructs assessed by each validated instrument, and no additional item-level selection procedures were performed. Although the included indicators originated from instruments with different internal structures, all network variables were conceptualized as construct-level representations of biological-related, psychological, or social factors. The objective was to examine how these constructs interact within a unified biopsychosocial system rather than to compare psychometric dimensions within a single instrument.

### 2.3 Data processing and analysis

Data were analyzed using R version 4.4.3. For descriptive purposes, original scale scores were summarized using means and standard deviations and compared across sex groups. These continuous scores were used only for descriptive statistical analyses and were not included in network estimation.

Prior to network estimation, questionnaire items were first recoded according to predefined binary criteria. Consistent with previous applications of the Ising model, questionnaire responses were recoded to represent the absence (0) versus presence (1) of clinically meaningful symptoms or characteristics prior to network estimation. For constructs without established clinical cutoffs, item responses were first dichotomized according to symptom presence versus absence, and construct-level binary indicators were subsequently derived based on the aggregated item information (van Borkulo et al., 2014). Items belonging to the same construct were subsequently aggregated, and the resulting construct-level indicators were transformed into binary variables using established clinical cutoffs, validated screening thresholds, or theoretically informed criteria (Wang Qi et al., 2006; Yanbo et al., 2018; Beck et al., 1988; Jianrong et al., 2002; Beck and Steer, 1996; Jianhui, 2002; Aloba et al., 2017; Li et al., 2007; Connor and Davidson, 2003; Jianxin, 2005; Kounios and Beeman, 2014; Thombs et al., 2007; Leung et al., 2010; Blumenthal et al., 1987). Detailed information regarding the derivation of each network node and the corresponding dichotomization criteria is provided in Supplementary Table S1. This procedure ensured that all network nodes were represented as construct-level binary indicators and could be analyzed within the Ising framework. Selected variables were reverse-coded where appropriate to ensure consistent interpretation across nodes.

Network nodes were derived according to the conceptual structure of the original instruments. For measures assessing a single overarching construct (e.g., anxiety, depression, childhood trauma, perceived stress, suicidal ideation, and suicidal behavior), total scores were used to represent the corresponding construct. For multidimensional instruments (e.g., Traditional Chinese Medicine constitution, emotion regulation difficulties, perceived social support, and dispositional insight), theoretically defined subscales were retained as separate network nodes to preserve construct-specific information.

The primary network was estimated using the Ising model implemented through the IsingFit estimator in the bootnet package. This approach employs logistic regression with eLasso regularization to estimate conditional associations among binary variables while reducing spurious connections. In the resulting network, nodes represented construct-level biopsychosocial indicators and edges represented conditional dependencies between node pairs after controlling for all remaining nodes.

To evaluate the robustness of the findings and assess the potential influence of dichotomization, a Gaussian Graphical Model (GGM) was additionally estimated as a sensitivity analysis using the EBICglasso procedure. The GGM was based on the original continuous indicators and enabled comparison of network structures across different modeling assumptions. Consistency between the Ising and GGM networks was interpreted as evidence supporting the robustness of the identified network architecture.

Expected Influence (EI) was used to evaluate node centrality within the network. Bridge centrality indices, including Bridge Expected Influence (BEI), were calculated to identify nodes that connected different biopsychosocial domains. Network accuracy and stability were assessed using nonparametric bootstrap procedures and case-dropping subset bootstrap analyses (Robinaugh et al., 2020; Qiang, 2018).

To examine the relative influence of individual nodes within the estimated network, Network Intervention Relative Ability (NIRA) analyses were conducted following the framework proposed by Lunansky et al. (2022). NIRA is a model-based perturbation method that evaluates how changes in a target node propagate through the estimated network.

NIRA analyses were based on the edge-weight matrix and threshold parameters obtained from the Ising model. For each biological-related and social node, aggravation and alleviation conditions were examined separately by increasing or decreasing the activation probability of the target node while holding all other model parameters constant. The resulting effects were propagated through the network, and changes in the aggregate activation of psychological-domain nodes were quantified.

Nodes producing larger changes in psychological-domain activation were considered relatively more influential within the estimated biopsychosocial network. Consistent with previous applications of NIRA, these analyses were intended to compare the relative influence of candidate nodes rather than estimate intervention efficacy or causal effects. Therefore, findings derived from NIRA should be interpreted as exploratory and hypothesis-generating.

## 3 Results

### 3.1 Descriptive statistics

As shown in Table 1, a total of 3,116 first-year university students were ultimately included in the study. The sample comprised 1,252 males (40.2%) and 1,864 females (59.8%), with an age range of 15–30 years and a mean age of 18.30 years (SD = 0.79). The majority of participants were of Han Chinese ethnicity (*n* = 2,956, 94.9%), while ethnic minority students accounted for 160 participants (5.1%). Regarding family structure, 735 participants (23.6%) were only children and 2,381 (76.4%) had siblings. Most participants were right-handed (*n* = 3,010, 96.6%), with 106 left-handed individuals (3.4%). All participants met the predefined inclusion criteria.

**Table 1:** Demographic characteristics of the participants.

Variable		*N*	Proportion (%)
Sex	Male	1252	40.2%
	Female	1864	59.8%
Ethnicity	Han Chinese	2956	94.9%
	Ethnic minority	160	5.1%
Only-child status	Only child	735	23.6%
	Non-only child	2381	76.4%
Handedness	Right-handed	3010	96.6%
	Left-handed	106	3.4%

Descriptive statistics for all biological-related, psychological, and social indicators derived from the original scales are presented in Supplementary Table S2. Detailed comparisons of each scale score between male and female students, including corresponding statistical tests, are reported in Supplementary Table S3.

Sex differences were examined using independent-samples t-tests, and corresponding effect sizes (Cohen’s d) are also reported. Several indicators showed significant sex differences, with female students generally reporting higher levels of psychological distress and perceived stress, whereas some constitution-related and psychosocial indicators demonstrated smaller sex-specific variations.

### 3.2 Network structure analysis

#### 3.2.1 Overall network

**Figure 1:**
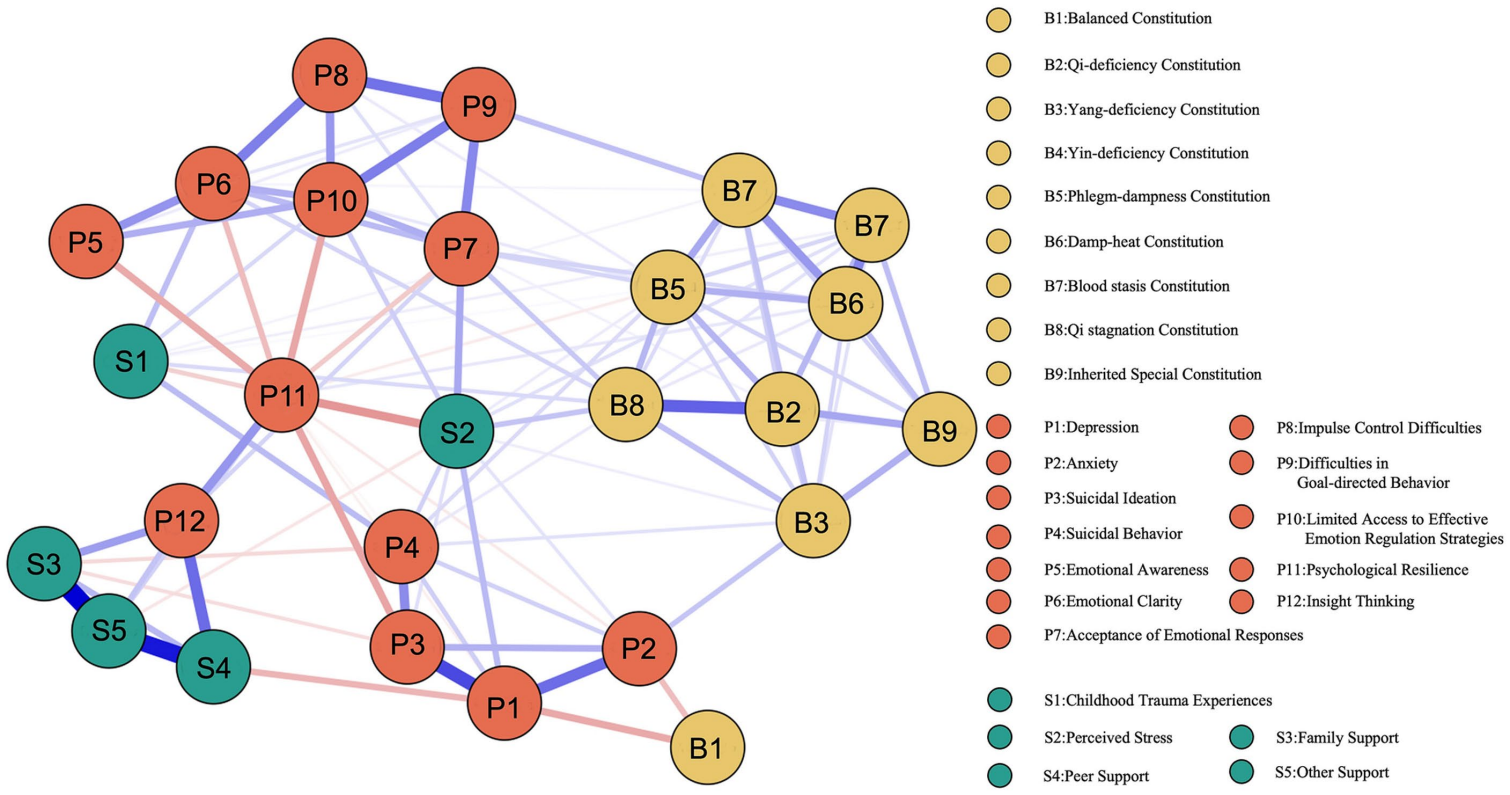
Biopsychosocial (BPS) network of first-year university students. Nodes represent construct-level indicators included in the network. Yellow nodes denote biological-related functional indicators (Traditional Chinese Medicine constitution types), green nodes denote social indicators (childhood trauma, perceived stress, and perceived social support), and red nodes denote psychological indicators (anxiety, depression, suicidal ideation, suicidal behavior, emotion regulation difficulty, resilience, and dispositional insight). Edges represent regularized conditional associations between node pairs after controlling for all remaining nodes in the network. Blue edges indicate positive associations, whereas red edges indicate negative associations. Thicker edges represent stronger associations. The spring layout places more strongly connected nodes closer together.

Figure 1 presents the biopsychosocial (BPS) network structure of first-year university students. Nodes are visualized as circular pie charts, with colors representing distinct domains (yellow for the biological-related network, red for the psychological network, and green for the social network). Blue edges indicate positive associations, whereas red dashed edges denote negative associations; edge thickness reflects the strength of the associations.

Within the biological-related network, the strongest undirected association was observed between Qi-deficiency constitution and blood-deficiency constitution. Within the psychological network, the strongest undirected edge was identified between depression and suicidal ideation. In the social network, family support showed a relatively strong association with other support within the perceived social support domain (r = 0.20); peer support was also strongly associated with other support. Across domains, the two bridge edges with the greatest weights were observed between depression in the psychological network and balanced constitution in the biological-related network, and between depression and peer support in the social network, both of which were negative associations.

#### 3.2.2 Centrality and bridge centrality

**Figure 2:**
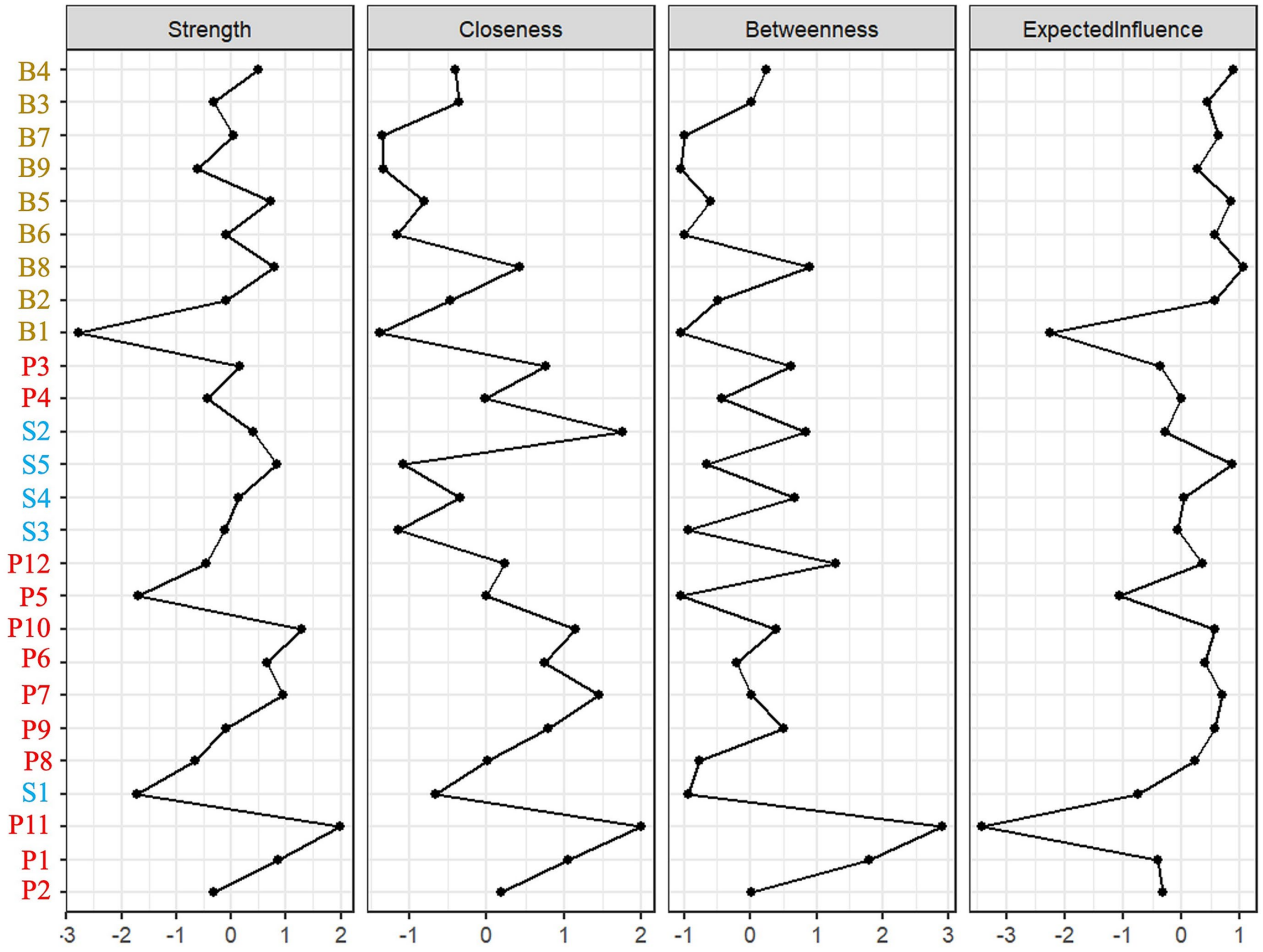
Centrality indices of nodes in the BPS network. The figure presents four commonly used centrality measures, including strength, closeness, betweenness, and expected influence (EI), for all nodes in the estimated BPS network. Values are standardized as z-scores to facilitate comparison across centrality metrics. Strength reflects the sum of the absolute edge weights directly connected to a node, indicating its overall level of connectivity within the network. Closeness reflects the average distance from a node to all other nodes and indicates how efficiently information may spread from that node throughout the network. Betweenness quantifies the extent to which a node lies on the shortest paths connecting other nodes and thus may function as an intermediary within the network. Expected influence (EI) represents the sum of all edge weights connected to a node while preserving the direction of positive and negative associations and was used as the primary indicator of node importance in the present study. Higher values indicate greater centrality and potential influence within the network.

After standardization of centrality indices, the results are shown in Figure 2. Strength centrality reflects the sum of a node’s direct connections to other nodes, indicating its overall importance within the network. Psychological resilience exhibited the highest strength centrality (Z = 1.99), suggesting that it was the most strongly connected node and may function as a core node in the network. Betweenness centrality captures the extent to which a node lies on the shortest paths between other nodes, reflecting its capacity to regulate information flow. Psychological resilience also demonstrated the highest betweenness centrality (Z = 2.91), underscoring its pivotal role as a hub within the network. Depression likewise showed a relatively high betweenness centrality (Z = 1.41), indicating its importance in linking multiple network components.

**Figure 3:**
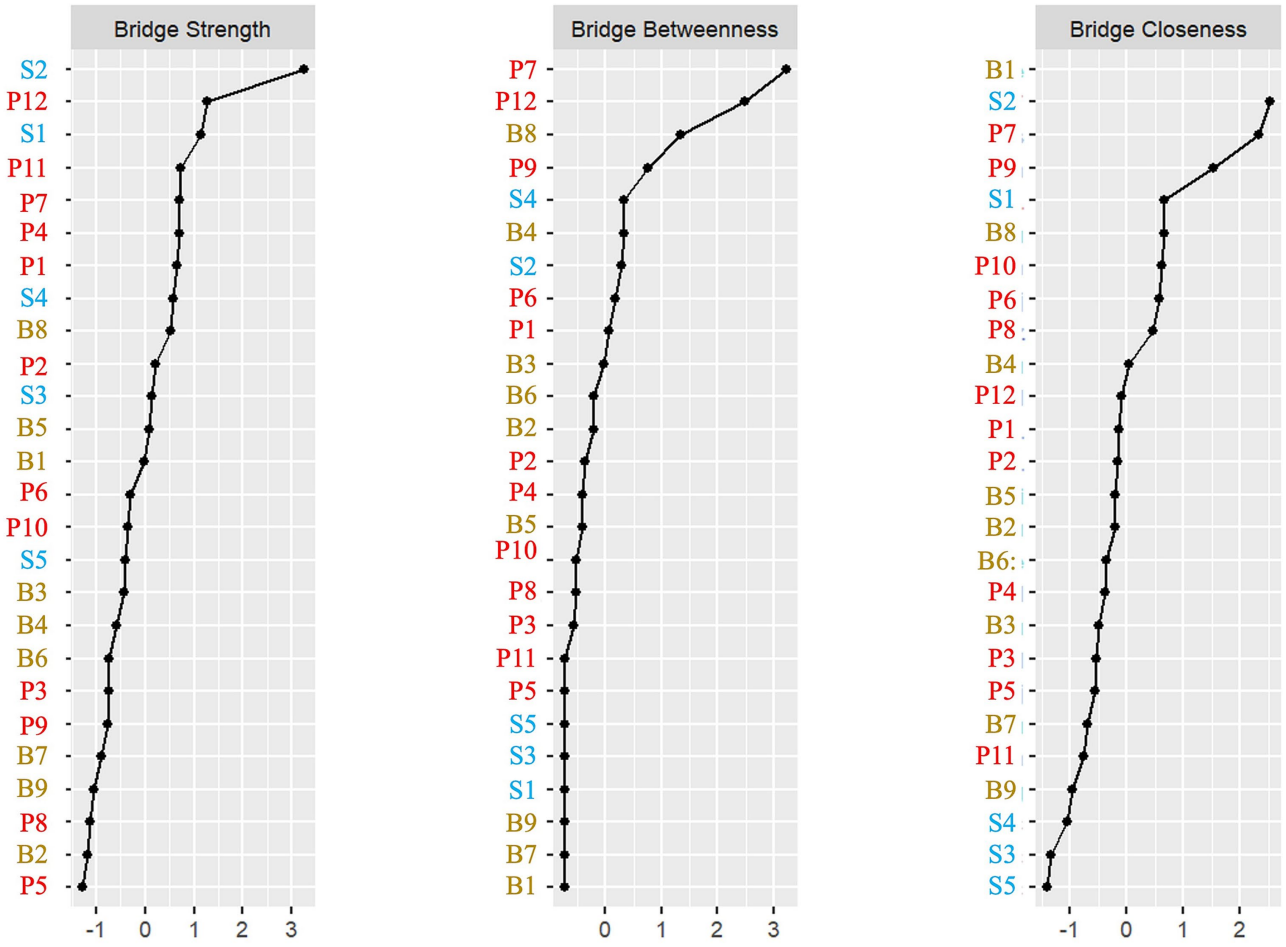
Bridge strength and bridge expected influence of Nodes. Bridge centrality metrics were calculated to identify nodes that connect different domains of the biopsychosocial network. The figure presents standardized values (z-scores) for Bridge Strength, Bridge Betweenness, and Bridge Closeness. Bridge Strength represents the sum of the absolute edge weights linking a node to nodes in other communities, indicating the extent to which a node directly connects different domains. Bridge Betweenness reflects the frequency with which a node lies on the shortest paths between nodes belonging to different communities, indicating its potential role in information transmission across domains. Bridge Closeness reflects the average distance from a node to nodes in other communities, with higher values indicating greater accessibility to other domains. Higher bridge centrality values suggest a greater potential for a node to facilitate interactions between biological-related, psychological, and social components of the network.

Bridge nodes were identified based on bridge strength centrality, which reflects the extent to which a node connects different subnetworks. As shown in Figure 3, perceived stress exhibited the highest bridge strength (bridge strength = 6.61; bridge expected influence = 4.04). Childhood trauma (bridge strength = 3.52; bridge expected influence = 2.38) and dispositional insight (bridge strength = 3.74; bridge expected influence = 3.74) also demonstrated strong bridging effects, highlighting their critical roles in cross-domain network connectivity.

#### 3.2.3 Network accuracy, stability and sensitivity analysis

**Figure 4:**
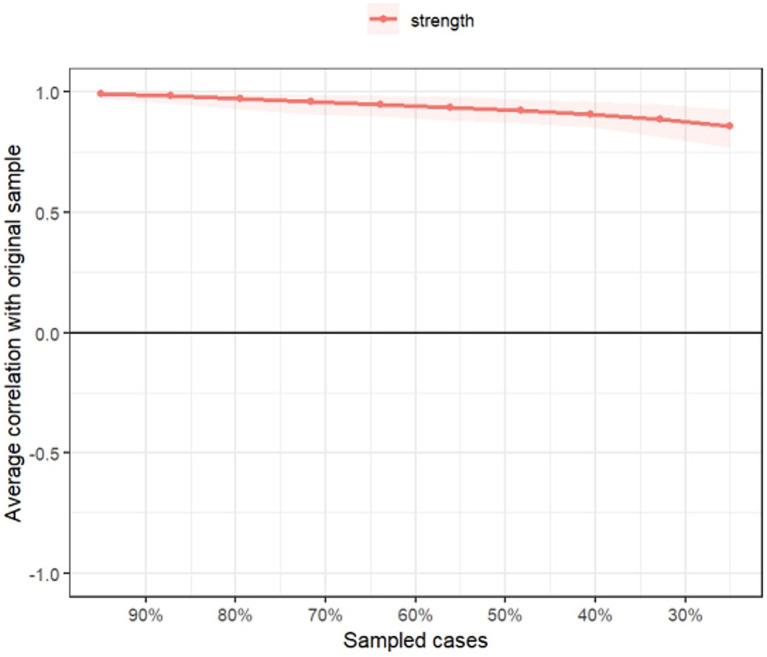
Stability analysis of strength centrality. The stability of node strength was evaluated using a case-dropping bootstrap procedure based on 5,000 resamples. The red line represents the correlation between strength centrality estimates obtained from subsets of the data and those derived from the original sample as increasing proportions of cases were removed. The *x*-axis indicates the proportion of cases retained, and the *y*-axis indicates the correlation with the original centrality estimates. Higher correlations reflect greater stability of the strength metric. The correlation stability (CS) coefficient was used to quantify robustness, with values above 0.50 indicating good stability of the centrality estimates.

Network accuracy and stability were evaluated using 5,000 bootstrap resamples, with results presented in Figure 4. Specifically, edge accuracy was assessed through bootstrap confidence intervals around edge weights, whereas network stability was evaluated using case-dropping bootstrap procedures for centrality and bridge-centrality metrics.

The analyses indicated satisfactory edge accuracy and overall network robustness (correlation stability [CS] coefficient = 0.75, 95% CI [0.672, 1.000]). The stability of strength centrality is shown in Figure 4. Additional analyses indicated that bridge strength (CS = 0.595) and bridge expected influence (CS = 0.75) also demonstrated acceptable stability according to recommended criteria.

To further assess network accuracy and robustness, an additional network was estimated using the Gaussian Graphical Model (GGM) and systematically compared with the Ising model–based network (Epskamp et al., 2018). The results demonstrated substantial concordance between the two modeling approaches with respect to core relational architecture and key node functions. Specifically, both the Ising and GGM networks consistently identified psychological resilience as the central hub node and perceived stress as a prominent bridge node.

Across both models, stable cross-domain associations were observed between Traditional Chinese Medicine (TCM) constitution types—particularly Qi stagnation—and psychological symptoms as well as social stressors. In addition, the two networks exhibited highly similar topological characteristics, including a predominance of positive associations and the clustering of strong connections within and across domains.

Bootstrap analyses further indicated acceptable stability across both models, with correlation stability (CS) coefficients of 0.75 for edge weights and strength centrality, and CS coefficients of 0.75 and 0.595 for bridge-related indices. These findings suggest that the overall network architecture and the identification of key nodes were robust to differences in model assumptions. Collectively, the results provide strong support for the reliability and generalizability of the study’s conclusions.

#### 3.2.4 NIRA simulation results

To identify biological-related and social factors that may exert relatively strong influences on psychological functioning within the estimated network, Network Intervention Relative Ability (NIRA) simulations were conducted. Specifically, each biological-related or social-domain node was subjected to simulated aggravation and alleviation perturbations, and the resulting changes in the aggregate activation of psychological-domain nodes were quantified.

**Figure 5:**
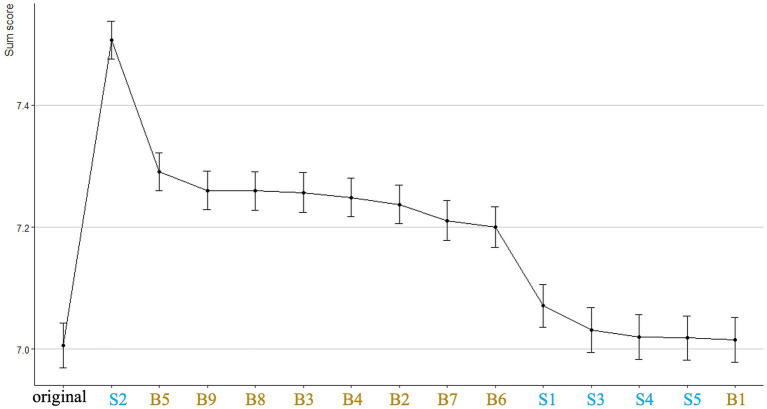
Results of aggravation intervention simulation. Results of the Network Intervention Relative Ability (NIRA) aggravation simulations. Each non-psychological node was individually subjected to a simulated aggravation perturbation, and the resulting changes in the psychological domain were quantified. The psychological domain comprised anxiety, depression, suicidal ideation, suicidal behavior, emotion regulation difficulty, resilience, and dispositional insight. Higher values indicate a greater increase in overall psychological-domain activation following simulated aggravation of the target node. These simulations represent model-based perturbations propagated through the estimated network structure and should not be interpreted as evidence of causal intervention effects.

Under the aggravation condition (Figure 5), perceived stress emerged as the most influential deterioration target, with simulated increases in stress producing the largest increase in overall psychological-domain activation. Aggravation of several Traditional Chinese Medicine (TCM) constitution types, including phlegm-dampness, inherited special constitution, Qi stagnation, Yin deficiency, Qi deficiency, and Yang deficiency, was also associated with increased activation of psychological-domain nodes.

**Figure 6:**
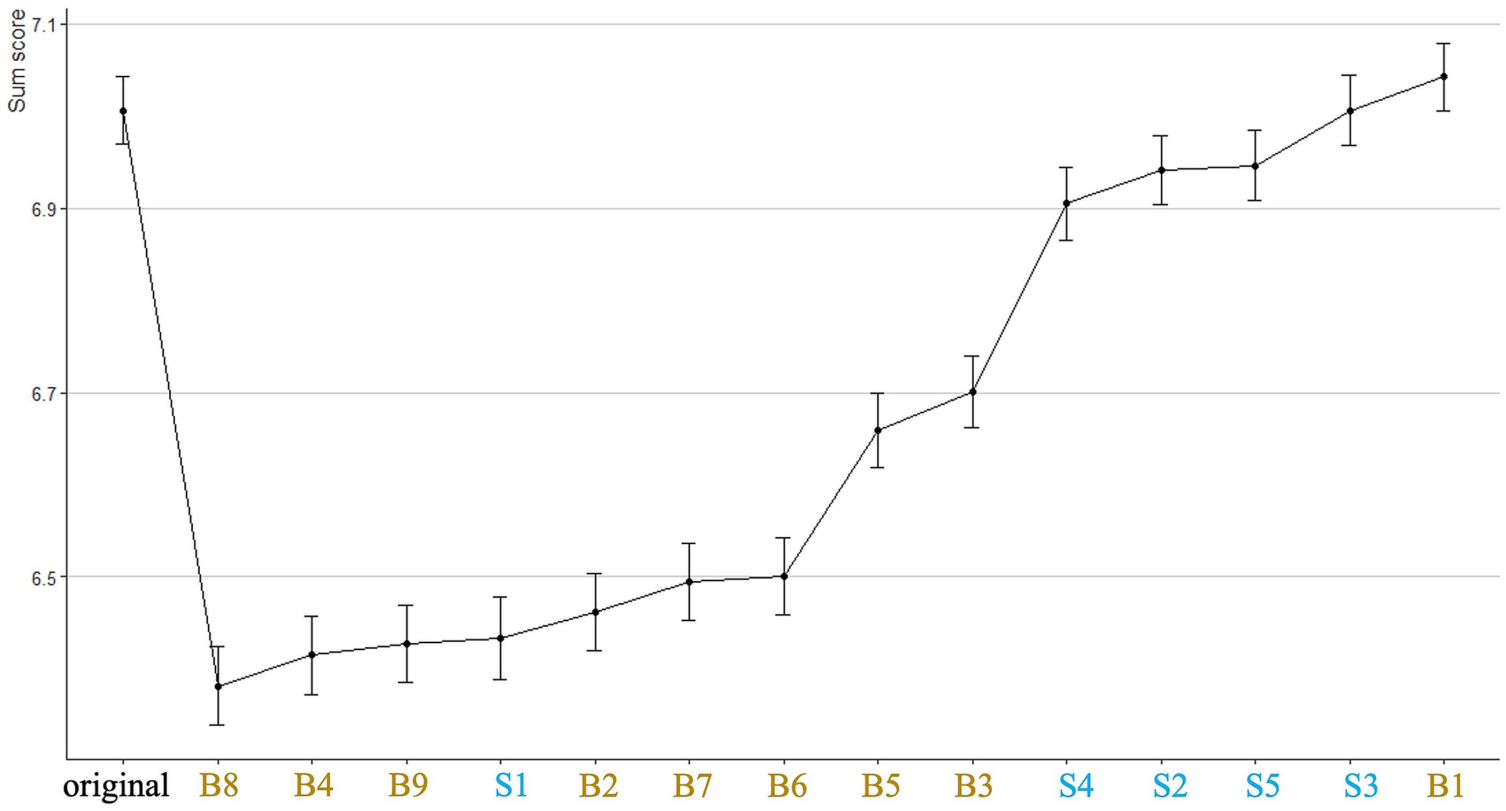
Results of alleviation intervention simulation. Results of the Network Intervention Relative Ability (NIRA) alleviation simulations. Each non-psychological node was individually subjected to a simulated alleviation perturbation, and the resulting changes in the psychological domain were quantified. The psychological domain comprised anxiety, depression, suicidal ideation, suicidal behavior, emotion regulation difficulty, resilience, and dispositional insight. Larger reductions in psychological-domain activation indicate greater relative influence of the perturbed node within the estimated network. Results reflect model-based simulations derived from the network structure and should be interpreted as indicators of relative network influence rather than causal intervention effects.

Under the alleviation condition (Figure 6), simulated improvement in Qi stagnation constitution produced the largest reduction in overall psychological-domain activation. In addition, alleviation of childhood trauma and improvement in several constitution types, including Yin deficiency, damp-heat, and Qi deficiency, were associated with substantial reductions in psychological-domain activation.

Taken together, these findings suggest that perceived stress, childhood trauma, and specific TCM constitution characteristics may occupy relatively influential positions within the estimated biopsychosocial network. However, because NIRA represents a model-based simulation procedure rather than an evaluation of real-world interventions, these results should be interpreted as indicators of relative network influence and potential intervention relevance rather than evidence of causal treatment effects.

### 3.3 Exploratory sex-specific network analyses

To explore potential sex-related differences in the organization of the biopsychosocial (BPS) network, separate networks were estimated for male (*n* = 1,252) and female (*n* = 1,864) freshmen. These analyses were conducted as exploratory subgroup investigations and should not be interpreted as formal tests of network differences.

Overall, both sex-specific networks showed structural patterns broadly consistent with those observed in the total sample. In both males and females, psychological resilience emerged as the most central node within the network, while perceived stress demonstrated the strongest bridge role linking social and psychological domains. These findings suggest that resilience and stress may represent robust components of the BPS system regardless of sex.

Several descriptive differences were nevertheless observed between the two networks. Compared with males, females exhibited a denser network structure, with a greater number of non-zero edges (190 vs. 160), suggesting stronger overall connectivity among biopsychosocial components. In addition, bridge indices indicated a more prominent role of perceived stress and childhood trauma in females, whereas constitution-related nodes appeared relatively more influential in males. These findings may suggest greater involvement of social stressors in the female network and greater prominence of constitution-related factors in the male network. However, because formal network comparison tests were not performed and network estimation is influenced by the distributional characteristics of the observed variables, these observations may reflect both variation in baseline construct distributions and potential differences in network organization. Therefore, the present findings should be interpreted as exploratory descriptive comparisons rather than definitive evidence of sex-specific biopsychosocial network structures.

Bootstrap analyses indicated acceptable stability for both networks, with strength centrality showing satisfactory correlation stability coefficients (CS = 0.75 in both sexes).

**Figure 7:**
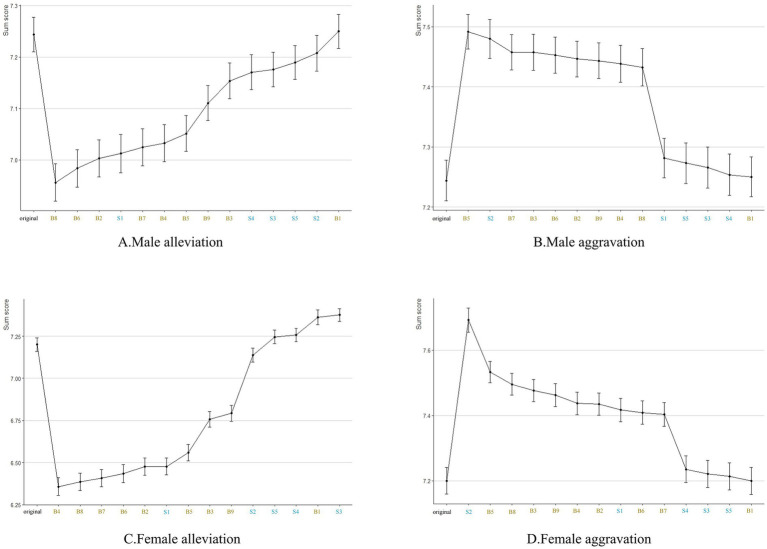
NIRA simulation results in male and female networks. Results of Network Intervention Relative Ability (NIRA) simulations conducted separately in male and female freshmen. Panel **(A)** presents alleviation simulations in the male network, Panel **(B)** presents aggravation simulations in the male network, Panel **(C)** presents alleviation simulations in the female network, and Panel **(D)** presents aggravation simulations in the female network. Each simulation was performed by individually modifying the activation probability of one biological-related or social node while keeping the remaining network structure unchanged. The *y*-axis (Sum Score) represents the simulated total score of the psychological-domain nodes following each perturbation. Lower values in the alleviation condition indicate greater predicted reductions in psychological symptom burden, whereas higher values in the aggravation condition indicate greater predicted increases in psychological symptom burden. Error bars represent the variability of the simulated psychological-domain scores across repeated response simulations. Nodes associated with larger changes in psychological-domain scores are interpreted as relatively influential components within the estimated biopsychosocial network. Given the model-based and cross-sectional nature of NIRA, these findings should be considered exploratory and hypothesis-generating rather than evidence of causal intervention effects.

Sex-related differences were also observed in the NIRA analyses (Figure 7). In males, constitution-related factors appeared to exert the strongest influence on psychological-domain activation, with alleviation of Qi stagnation constitution producing the largest reduction and aggravation of phlegm-dampness constitution producing the largest increase in activation. In contrast, in females, alleviation of Yin-deficiency constitution yielded the greatest reduction in activation, whereas aggravation of perceived stress produced the strongest increase. These findings suggest that constitution-related factors may play a relatively larger role in the male network, whereas stress-related processes may be more influential in the female network. Consistent with the overall-sample findings, perceived stress and constitution-related characteristics emerged as potentially influential components within the modeled system. Nevertheless, given the cross-sectional and model-based nature of NIRA, as well as the possibility that sex-specific network differences may partly reflect differences in baseline variable distributions, these findings should be regarded as exploratory and hypothesis-generating rather than evidence of causal intervention effects or definitive sex-specific intervention targets.

## 4 Discussion

Grounded in the biopsychosocial (BPS) framework, this study applied network analysis to examine the complex interactions among biological-related, psychological, and social factors in university freshmen. The findings suggest that psychological resilience occupies a central position within the network, whereas perceived stress plays a key bridging role linking different domains. Simulation analyses further indicate that changes in these components may be associated with variations in overall network activation. However, given the cross-sectional design and the model-based nature of the simulations, these findings should be interpreted as associative rather than causal.

### 4.1 Psychological resilience: protective functions and the enhancement of well-being

Across both the overall biopsychosocial (BPS) network and the gender-specific networks, psychological resilience consistently exhibited the highest strength and betweenness centrality, indicating its stable and central position within the network structure. This pattern suggests that psychological resilience is highly connected to multiple biological-related, psychological, and social nodes and may function as a key hub facilitating interactions across domains.

Previous studies have shown that psychological resilience is associated with reduced depressive symptoms under stress, potentially through the adoption of more adaptive coping strategies (Qiang, 2018). In addition, resilience has been linked to enhanced well-being via increased positive emotional experiences, suggesting that it may operate not only as a relatively stable trait but also as a dynamic capacity related to emotion regulation processes (Yong and Zhenhong, 2013).

Consistent with these findings, the present study indicates that psychological resilience occupies a central position within the psychological domain and is connected to perceived stress, childhood trauma, and Traditional Chinese Medicine (TCM) constitution through multiple bridging pathways, highlighting its integrative role within the BPS framework.

Moreover, psychological resilience demonstrated consistently high centrality indices across both male and female networks, suggesting its robustness and potential cross-gender relevance among university freshmen. From a network perspective, this pattern highlights psychological resilience as a node that may play an important role within the system.

Importantly, it is necessary to distinguish between structural importance and potential system influence. Although centrality indices identify nodes that are highly connected within the network, they do not necessarily imply that modifying these nodes would result in the greatest changes at the system level. Therefore, simulation analyses were conducted to complement the structural findings and to explore potential system responses to perturbations in node activation.

### 4.2 Depression risk and the protective role of social support systems

The results indicated that depression exhibited the strongest undirected association with suicidal ideation within the psychological domain, consistent with prior symptom network research and supporting its central position within suicide-related networks (Robinaugh et al., 2020; de Beurs et al., 2017).

Furthermore, depression demonstrated significant negative associations, reflected in the two highest-weight cross-domain bridging edges, with Balanced Constitution in the biological-related domain and peer support in the social domain. From a network perspective, these associations may reflect potential pathways through which depressive symptoms are linked to broader biopsychosocial components.

At the biological-related level, Balanced Constitution is conceptualized in Traditional Chinese Medicine as a state characterized by relative physiological and psychosomatic balance, including coordinated yin–yang dynamics and stable functional regulation (Wang Qi et al., 2006). The observed negative association between depression and Balanced Constitution may suggest that deviations from this balanced state are linked to higher levels of depressive symptoms.

At the social level, peer support—one of the most salient sources of support during the freshman period—also exhibited a negative bridging association with depression, consistent with the social support buffering hypothesis (Cohen and Wills, 1985). Previous studies have reported that lower levels of peer support are associated with increased depressive symptoms and stronger associations between depression and suicidal ideation (Kleiman and Liu, 2013). Peer support may provide emotional resources, instrumental assistance, and adaptive coping opportunities, thereby being linked to more favorable psychological outcomes among university students. For example, Zhou Li and colleagues found that higher levels of peer support were associated with lower levels of depressive symptoms under conditions of cyberbullying (Li et al., 2024).

Taken together, these findings highlight the potential role of peer support and biological-related constitutional status as important components within the network. However, these associations should be interpreted cautiously, as they do not imply directional or causal relationships but rather reflect system-level connections within the modeled network.

### 4.3 The negative impact of stress: gender differences from perception to neural foundations

Across both the overall network and the gender-stratified networks, perceived stress consistently exhibited the highest bridge strength and bridge expected influence, indicating its prominent role in connecting different domains within the network. In the simulation analyses, increased activation of perceived stress was associated with the largest increase in overall psychological network activation, further suggesting its potential importance in system-level dynamics.

In previous research, stress has been conceptualized as a state involving psychological strain accompanied by physiological tension in response to negative life events (Hanteng, 2003). Empirical studies have also indicated that stress is associated with both constitutional characteristics and psychological functioning, potentially through mediating processes such as psychological flexibility (Haidan et al., 2024). Consistent with this evidence, the present findings suggest that perceived stress may function as a key bridging component linking social environmental factors with psychological responses within the network.

Gender comparisons indicated that stress-related bridging indices and simulation-based associations were more pronounced in the female network than in the male network. This pattern may reflect gender-related differences in stress perception and emotional processing. Prior studies have shown that females tend to report greater sensitivity to social evaluation, interpersonal dynamics, and emotional cues, and may exhibit higher levels of emotional reactivity and ruminative cognition under stress (Parker and Brotchie, 2010; Matud, 2004). Neuroimaging evidence further suggests that, under negative emotional conditions, females may show relatively greater activation in self-referential processing regions, such as the cortical midline structures and the precuneus, whereas males may exhibit relatively greater activation under positive emotional conditions (Lanlan, 2014).

Taken together, these psychological and neurobiological patterns may provide a potential explanation for observed gender differences in depression. However, these interpretations should be considered within the context of associative and model-based findings. From a network perspective, stress-related nodes may represent components with relatively strong cross-domain connectivity, highlighting their potential relevance within the overall system. This interpretation is also supported, to some extent, by recent network studies in university student populations (Sun et al., 2024; Sun et al., 2025). For example, previous research has shown that affective and stress-related components, along with stress management, can occupy important positions in networks linking anxiety, depression, sleep problems, and health-promoting lifestyles. Other work has similarly identified fatigue, guilt, and difficulty relaxing as central symptoms in depression–anxiety networks among medical students (Sun et al., 2024; Sun et al., 2025). Together, these findings reinforce the view that stress- and depression-related components may play structurally important roles in student mental health networks. The present study builds on this line of research by further incorporating biological-related functional indicators within a biopsychosocial framework.

### 4.4 Gender differences in psychological network regulation based on traditional Chinese medicine constitution

It should be noted that TCM constitution, as assessed in this study, does not represent direct biological measurements but rather reflects functional and phenomenological states that may be associated with underlying physiological processes. Accordingly, its classification within the biological domain should be interpreted as a theoretical extension within the BPS framework rather than a strict biological indicator. Within this framework, such constructs may be understood as relatively stable functional patterns associated with physiological and psychosomatic processes.

A key contribution of the present study lies in integrating Traditional Chinese Medicine (TCM) constitution into network analysis and simulation-based frameworks, thereby enabling the examination of how different constitution types are associated with variations in network structure and activation. Simulation analyses suggested that reduced activation of Qi stagnation constitution was associated with the largest decrease in overall psychological network activation in the overall and male-specific networks, whereas reduced activation of Yin-deficiency constitution was associated with the largest decrease in the female network.

From the perspective of TCM theory, these patterns may be broadly consistent with traditional conceptualizations. Qi stagnation constitution is typically characterized by emotional constraint and dysregulation of qi flow and has been linked to depressive mood, irritability, and anxiety-related symptoms (Wang, 1995). Empirical studies have further reported that individuals with Qi stagnation constitution tend to show greater vulnerability in emotion regulation and stress reactivity (Xinzhu and Yuanchun, 2024; Zhang et al., 2025). These associations may help to contextualize the observed network patterns in the male subgroup, although such interpretations remain tentative within a cross-sectional and model-based framework.

In contrast, classical TCM literature describes women as being more closely associated with blood and yin-related processes, which has been interpreted as a predisposition toward patterns resembling Yin deficiency. Yin-deficiency constitution has been associated with symptoms such as irritability, sleep disturbances, and affective instability. These characteristics are broadly consistent with findings from contemporary psychological research indicating that emotional dysregulation among females may be associated with greater emotional reactivity, sleep-related disturbances, and stress sensitivity. However, the present findings do not provide evidence regarding the biological mechanisms underlying these associations (Albert, 2015).

From a network perspective, these findings suggest that TCM constitution may function as a component associated with variations in network organization, rather than as a fixed biological determinant. The identified patterns highlight constitution-related nodes as potentially influential within the modeled system. However, these results should be interpreted as simulation-based and hypothesis-generating, and do not imply that modifying these nodes would necessarily lead to corresponding changes in real-world psychological outcomes.

Beyond the interpretation of specific findings, the present study raises a broader theoretical question regarding the integration of biological-related, psychological, and social indicators within a single network framework. Contemporary network theory suggests that network models are not restricted to a particular level of explanation and may incorporate biological, psychological, and environmental components within a common system. Rather than assuming that all variables reflect a single latent construct, network approaches conceptualize mental health as emerging from interactions among multiple components that may originate from different explanatory levels. In this sense, the present biopsychosocial network was intended to examine how diverse risk and protective factors are conditionally associated within a broader mental health system. Consistent with Borsboom’s network theory, such networks can be viewed as an organizing framework that facilitates the integration of biological, psychological, and social perspectives while remaining agnostic regarding the precise mechanisms underlying observed associations. Accordingly, the edges identified in the present study should be interpreted as conditional associations among theoretically relevant components rather than as direct evidence of causal, biological, or psychological mechanisms.

Furthermore, although several descriptive differences were observed between the male and female networks, these findings should be interpreted cautiously. Because network estimation depends on the distributional characteristics of the included variables, observed differences in connectivity patterns and node importance may reflect both variation in baseline construct distributions and potential differences in network organization. Future studies using formal network comparison procedures are needed to further clarify the sources of observed sex-related differences.

### 4.5 Limitations and future directions

Several limitations of the present study should be acknowledged.

First, the cross-sectional design precludes conclusions regarding temporal ordering or causal relationships among network components. Although NIRA was used to examine the relative propagation of perturbations within the estimated network structure, these analyses represent model-based simulations rather than evidence of causal effects or actual intervention efficacy. Accordingly, the findings should be interpreted as exploratory and hypothesis-generating. Future longitudinal, intensive longitudinal, and experimental studies are needed to clarify the temporal dynamics and potential causal relevance of influential network components.

Second, although all network nodes were ultimately represented as construct-level binary indicators, the original constructs were derived from instruments with different psychometric structures and theoretical backgrounds. Consequently, some residual differences in construct specificity may remain. Although contemporary network theory permits the integration of heterogeneous components within a common network framework (Borsboom, 2017; Cai et al., 2022; Feng et al., 2025), the interpretation of cross-domain associations remains partly theory-dependent and therefore warrants cautious interpretation. Furthermore, the sex-specific analyses conducted in this study were exploratory in nature. Because network estimation is influenced by the distributional characteristics of the included variables, observed differences between male and female networks may reflect both variation in baseline construct distributions and potential differences in network organization. Formal network comparison procedures were not conducted in the present study; therefore, the sources of the observed sex-related differences remain uncertain and should be interpreted cautiously. Furthermore, the present study focused on node-level centrality and bridge characteristics and did not examine potential modular structures within the network. Recent methodological developments have suggested that community detection and module-level control analyses may provide additional insights into the organization and regulation of psychopathology networks (Pan et al., 2024). Future studies may therefore benefit from integrating multilevel or hierarchical network approaches with community detection methods to further investigate the organization of biopsychosocial systems (Blanken et al., 2018).

Third, the sample was drawn exclusively from universities in South China, which may limit the generalizability of the findings to other populations and contexts. Replication using more geographically, culturally, and educationally diverse samples is needed to evaluate the stability and reproducibility of the observed network structure.

Finally, TCM constitution was assessed using a self-report instrument and was conceptualized as a biological-related functional indicator rather than a direct biological measure. Although this classification was theoretically motivated, alternative conceptualizations may also be plausible. Furthermore, some constructs included in the network are conceptually related and may share overlapping variance despite redundancy analyses indicating no need for node removal. Therefore, the findings should be interpreted primarily in terms of the overall organization of the biopsychosocial network rather than overly fine distinctions between highly related constructs. Future studies integrating constitution-related measures with objective physiological, behavioral, and biological indicators may help further clarify these relationships.

## 5 Conclusion

By integrating the biopsychosocial (BPS) framework with network theory, this study constructed a multidimensional interactive mental health network among Chinese first-year university students and conducted simulation analyses using the Node Identify via Recursive Graphs (NIRA) algorithm. The findings indicate that psychological resilience occupies a central position within the network, whereas perceived stress appears to function as a key bridging component linking biopsychosocial domains.

Simulation results further suggested that increased activation of perceived stress was associated with the largest increase in overall psychological network activation, while reduced activation of Qi stagnation constitution was associated with the largest decrease. These patterns highlight components that may be particularly relevant within the network structure.

Overall, the present findings provide a system-level characterization of freshmen’s mental health. However, the results should be interpreted as model-based and exploratory, offering a basis for hypothesis generation rather than direct implications for causal mechanisms or real-world interventions.

## Data Availability

The datasets presented in this article are not readily available because data was collected through questionnaires and other forms, and the principle of confidentiality was promised to the subjects. Requests to access the datasets should be directed to Wenjie Zhang, mankit_psy1029@163.com.

## References

[ref1] Albert P. R. (2015). Why is depression more prevalent in women? J. Psychiatry Neurosci. 40, 219–221. doi: 10.1503/jpn.150205, 26107348 PMC4478054

[ref2] Aloba O. Ojeleye O. Aloba T. (2017). The psychometric characteristics of the 4-item suicidal behaviors questionnaire-revised (SBQ-R) as a screening tool in a non-clinical sample of Nigerian university students. Asian J. Psychiatr. 26, 46–51. doi: 10.1016/j.ajp.2017.01.017, 28483090

[ref3] Arnett J. J. (2004). Emerging Adulthood: The Winding Road from the Late Teens through the Twenties. New York, NY: Oxford University Press.

[ref4] Auerbach R. P. Mortier P. Bruffaerts R. Alonso J. Benjet C. Cuijpers P. . (2018). WHO world mental health surveys international college student project: prevalence and distribution of mental disorders. J. Abnorm. Psychol. 127, 623–638. doi: 10.1037/abn0000362, 30211576 PMC6193834

[ref5] Beck A. T. Epstein N. Brown G. Steer R. A. (1988). An inventory for measuring clinical anxiety: psychometric properties. J. Consult. Clin. Psychol. 56, 893–897. doi: 10.1037//0022-006x.56.6.893, 3204199

[ref6] Beck A. T. Steer R. A. (1996). Beck Depression Inventory: Manual. San Antonio: The Psychological Corporation Harcourt Brace Jovanovich.

[ref7] Blanken T. F. Deserno M. K. Dalege J. Borsboom D. Blanken P. Kerkhof G. A. . (2018). The role of stabilizing and communicating symptoms given overlapping communities in psychopathology networks. Sci. Rep. 8:5854. doi: 10.1038/s41598-018-24224-2, 29643399 PMC5895626

[ref8] Blumenthal J. A. Burg M. M. Barefoot J. Williams R. B. Haney T. Zimet G. (1987). Social support, type a behavior, and coronary artery disease. Psychosom. Med. 49, 331–340. doi: 10.1097/00006842-198707000-00002 3615762

[ref9] Borrell-Carrió F. Suchman A. L. Epstein R. M. (2004). The biopsychosocial model 25 years later: principles, practice, and scientific inquiry. Ann. Fam. Med. 2, 576–582. doi: 10.1370/afm.245, 15576544 PMC1466742

[ref10] Borsboom D. (2017). A network theory of mental disorders. World Psychiatry 16, 5–13. doi: 10.1002/wps.20375, 28127906 PMC5269502

[ref11] Cai H. Bai W. Liu H. Chen X. Qi H. Liu R. . (2022). Network analysis of depressive and anxiety symptoms in adolescents during the later stage of the COVID-19 pandemic. Transl. Psychiatry 12:98. doi: 10.1038/s41398-022-01838-9, 35273161 PMC8907388

[ref12] Cohen S. Wills T. (1985). Stress, social support, and the buffering hypothesis. Psychol. Bull. 98, 310–357. doi: 10.1037/0033-2909.98.2.310 3901065

[ref13] Connor K. M. Davidson J. R. (2003). Development of a new resilience scale: the Connor-Davidson resilience scale (CD-RISC). Depress. Anxiety 18, 76–82. doi: 10.1002/da.10113, 12964174

[ref14] Credé M. Niehorster S. (2012). Adjustment to college as measured by the student adaptation to college questionnaire: a quantitative review of its structure and relationships with correlates and consequences. Educ. Psychol. Rev. 24, 133–165. doi: 10.1007/s10648-011-9184-5

[ref15] de Beurs D. P. van Borkulo C. D. O'Connor R. C. (2017). Association between suicidal symptoms and repeat suicidal behaviour within a sample of hospital-treated suicide attempters. BJPsych Open 3, 120–126. doi: 10.1192/bjpo.bp.116.004275, 28507771 PMC5415676

[ref16] Engel G. L. (1977). The need for a new medical model: a challenge for biomedicine. Science 196, 129–136. doi: 10.1126/science.847460, 847460

[ref17] Epskamp S. Borsboom D. Fried E. I. (2018). Estimating psychological networks and their accuracy: a tutorial paper. Behav. Res. Methods 50, 195–212. doi: 10.3758/s13428-017-0862-1, 28342071 PMC5809547

[ref18] Feng R. Zhang Q. Wang L. Lei J. Liu X. Chen Z. . (2025). Network analysis of the relationship between negative life events and depressive symptoms among adolescents: a comparison between males and females. Front. Pediatr. 13:1624250. doi: 10.3389/fped.2025.1624250, 41346666 PMC12673483

[ref19] Haidan L. Xiao M. Wenbo W. Zhen L. Yanli J. Rongjuan G. (2024). Mediating effect of stress perception between TCM constitution and mental health. Liaoning J. Tradit. Chin. Med. 51, 6–10. doi: 10.13192/j.issn.1000-1719.2024.11.002

[ref20] Hanteng Y. T. H. (2003). An epidemiological study on stress among urban residents in social transition period. Chin. J. Epidemiol. 9, 11–15. doi: 10.3760/cma.j.issn.1674-6554.2007.04.017 14521764

[ref21] Huang H. Song Q. Chen J. Zeng Y. Wang W. Jiao B. . (2022). The role of Qi-stagnation constitution and emotion regulation in the association between childhood maltreatment and depression in Chinese college students. Front. Psych. 13:825198. doi: 10.3389/fpsyt.2022.825198, 35599766 PMC9114459

[ref22] Jianhui X. (2002). Preliminary formulation of the self-assessment scale of suicide thoughts. J. Clin. Psychiatry 2, 100–102. doi: 10.3969/j.issn.1005-3220.2002.02.030

[ref23] Jianrong Z. Chirong H. Jiejing H. Xiangquan Z. Debao W. Shuyi Z. . (2002). A study of psychometric properties, normative scores and factor structure of Beck anxiety inventory Chinese version. Chin. J. Clin. Psych. 1, 4–6. doi: 10.16128/j.cnki.1005-3611.2002.01.002

[ref24] Jianxin Y. X. Z. (2005) Application of Connor-Davidson toughness table (CD-RISC) in mainland China. The 10th National Academic Conference on Psychology, Shanghai, China

[ref25] Jinhe W. M. L. D. D. Miao C. J. Q. (2024). Relationship between qi stagnation constitution and anxiety/depressionin 10,325 junior high school students, and in which the mediating effectof sleep duration. J. Beijing Univ. Traditional Chinese Med. 47, 1457–1465. doi: 10.3969/j.issn.1006-2157.2024.10.016

[ref26] Kleiman E. M. Liu R. T. (2013). Social support as a protective factor in suicide: findings from two nationally representative samples. J. Affect. Disord. 150, 540–545. doi: 10.1016/j.jad.2013.01.033, 23466401 PMC3683363

[ref27] Kounios J. Beeman M. (2014). The cognitive neuroscience of insight. Annu. Rev. Psychol. 65, 71–93. doi: 10.1146/annurev-psych-010213-115154, 24405359

[ref28] Koushik M. Veeramani P. Vaishali L. P. M. Charumathi B. Jain T. (2025). Suicidal ideation and its associated risk factors among students of a medical college in Chennai-a cross-sectional study. J. Family Med. Prim. Care 14, 62–69. doi: 10.4103/jfmpc.jfmpc_446_24, 39989591 PMC11844983

[ref29] Lanlan Z. (2014) Gender Differences in cerebral Activity during Emotion Perception of five Arousal Levels: An event-Related fMRI Study [硕士, Shanghai, China: Shanghai University of Sport]. Available online at: https://kns.cnki.net/kcms2/article/abstract?v=1UNTTfPTmO_TUNmKrw0k4cnzGR4Xw4jLrKb9YH0qiKRgAcbM_X-Holaw-xBqBMURyWEgx-gAupunmaBod_2ujxT1I5Ebd00_Aejj492nNvbNpo0Kdcl3I_4t5fOEM9Dqn5vhNOgDKjzNYAmzj8QF6CN-mg8_r8BLrrb_QuartO6pRKv6SYXcVrLxvi2UiHeS&uniplatform=NZKPT&language=CHS

[ref30] Leung D. Y. Lam T. H. Chan S. S. (2010). Three versions of perceived stress scale: validation in a sample of Chinese cardiac patients who smoke. BMC Public Health 10:513. doi: 10.1186/1471-2458-10-513, 20735860 PMC2939644

[ref31] Li W. Hengchao L. Wei D. Zhongquan L. (2007). Test of difficulties in emotion regulation scale in Chinese people. Chin. J. Health Psychol. 4, 336–340. doi: 10.13342/j.cnki.cjhp.2007.04.020

[ref32] Li Z. Hongxia W. Jingyu G. Li L. (2024). Cyberbullying victimization and depression among college students:the moderating roles of psychological capital and peer support. J. Psychol. Sci. 47, 981–989. doi: 10.16719/j.cnki.1671-6981.20240427

[ref33] Liu J. Yan F. Ma X. Guo H. L. Tang Y. L. Rakofsky J. J. . (2016). Prevalence of major depressive disorder and socio-demographic correlates: results of a representative household epidemiological survey in Beijing, China (vol 179, pg 74, 2015) [correction]. J. Affect. Disord. 197, 8–8. doi: 10.1016/j.jad.2016.01.044, 25845752 PMC7127303

[ref34] Lunansky G. Naberman J. van Borkulo C. D. Chen C. Wang L. Borsboom D. (2022). Intervening on psychopathology networks: evaluating intervention targets through simulations. Methods 204, 29–37. doi: 10.1016/j.ymeth.2021.11.006, 34793976

[ref35] Matud M. P. (2004). Gender differences in stress and coping styles. Pers. Individ. Dif. 37, 1401–1415. doi: 10.1016/j.paid.2004.01.010

[ref36] Oliveira S. Macarico T. Pacheco R. Janeiro I. Marques-Pinto A. (2025). Mind the (social and emotional competence) gap to support higher education students' well-being: psychometric properties of the SECAB-A(S). Eur. J. Investig. Health Psychol. Educ. 15:162. doi: 10.3390/ejihpe15080162, 40863284 PMC12385931

[ref37] Ouyang H. Wu L. Yan W. Si K. Lv H. Zhan J. . (2024). Network analysis of the comorbidity between post-traumatic stress, depression and anxiety symptoms among frontline healthcare workers during the COVID-19 pandemic. Ther. Adv. Psychopharmacol. 14:20451253241243292. doi: 10.1177/20451253241243292, 38644941 PMC11032008

[ref38] Pan C. Zhang Q. Zhu Y. Kong S. Liu J. Zhang C. . (2024). Module control of network analysis in psychopathology. iScience 27:110302. doi: 10.1016/j.isci.2024.110302, 39045106 PMC11263636

[ref39] Papadimitriou G. (2017). The "biopsychosocial model": 40 years of application in psychiatry. Psychiatriki 28, 107–110. doi: 10.22365/jpsych.2017.282.107 28686557

[ref40] Park H. Noh K. Choi S. Min J. J. (2023). Social support as a moderator between resilience and psychological distress among Korean Americans perceiving racial discrimination during COVID-19: an exploratory application of a moderated mediation model. Int. J. Intercult. Relat. 95:101815. doi: 10.1016/j.ijintrel.2023.101815

[ref41] Parker G. Brotchie H. (2010). Gender differences in depression. Int. Rev. Psychiatry 22, 429–436. doi: 10.3109/09540261.2010.492391, 21047157

[ref43] Qiang Y. (2018). The effect of perceived stress on college students' depression:moderated mediating effect. Psychol. Dev. Educ. 34, 497–503. doi: 10.16187/j.cnki.issn1001-4918.2018.04.14

[ref44] Robinaugh D. J. Hoekstra R. H. A. Toner E. R. Borsboom D. (2020). The network approach to psychopathology: a review of the literature 2008-2018 and an agenda for future research. Psychol. Med. 50, 353–366. doi: 10.1017/S0033291719003404, 31875792 PMC7334828

[ref45] Sun C. Han J. Zhu Z. Zhang Q. Wang P. Zhang P. . (2025). Network analysis of comorbid depression and anxiety and their associations with academic engagement among medical students in the post-peak COVID-19 period in China. BMC Psychol. 13:838. doi: 10.1186/s40359-025-03181-2, 40713767 PMC12297837

[ref46] Sun C. Zhu Z. Zhang P. Wang L. Zhang Q. Guo Y. . (2024). Exploring the interconnections of anxiety, depression, sleep problems and health-promoting lifestyles among Chinese university students: a comprehensive network approach. Front. Psych. 15:1402680. doi: 10.3389/fpsyt.2024.1402680, 39077626 PMC11284064

[ref47] Thombs B. D. Lewis C. Bernstein D. P. Medrano M. A. Hatch J. P. (2007). An evaluation of the measurement equivalence of the childhood trauma questionnaire--short form across gender and race in a sample of drug-abusing adults. J. Psychosom. Res. 63, 391–398. doi: 10.1016/j.jpsychores.2007.04.010, 17905047

[ref48] van Borkulo C. D. Borsboom D. Epskamp S. Blanken T. F. Boschloo L. Schoevers R. A. . (2014). A new method for constructing networks from binary data. Sci. Rep. 4:5918. doi: 10.1038/srep05918, 25082149 PMC4118196

[ref9001] Wang W. (1995). Traditional Chinese Medicine Constitution. Beijing, China: China Medical Science Press.

[ref49] Wang Qi Z. Y. Hesheng X. Xiao L. (2006). Preliminary compilation of the quality table of traditional Chinese medicine. Clinical rehabilitation in China 3, 12–14.

[ref50] Xinzhu W. Yuanchun H. (2024). Qi stagnation and qi deficiency are associated with depression in college students. Front. Public Health 12, 1444237–1444237. doi: 10.3389/fpubh.2024.1444237, 39220450 PMC11362030

[ref51] Yanbo Z. Qi W. Huimei S. Xiaohan Y. (2018). Formulation and evaluation on short version of Chinese medical constitution questionnaire with 30 items. J. Tradit. Chin. Med. 59, 1554–1559. doi: 10.13288/j.11-2166/r.2018.18.006

[ref52] Yong W. Zhenhong W. (2013). Resilience of college students and the relations of resilience to positive emotion and to subjective well-being. Psychol. Dev. Educ. 29, 94–100. doi: 10.16187/j.cnki.issn1001-4918.2013.01.008

[ref53] Zhang H. Chen J. Chen J. Liu Y. Yu J. Wang J. . (2025). Associations between qi stagnation constitution, suboptimal health status, and lifestyle factors in southern China: a population-based cross-sectional study. J. Tradit. Chin. Med. Sci. 12, 521–530. doi: 10.1016/j.Jtcms.2025.09.002

[ref54] Zhang Mengmeng Q. Z. (2024). Research on the early detection and scientific intervention mechanism of psychological problems of college students from the perspective of positive psychology. J. Lvliang Univ. 14, 73–78.

