# Supplementary material for: Pathways for the precise prevention and improvement of mental health among university freshmen: a network analysis and simulated intervention study based on the biopsychosocial model
Source: Front Psychol. 2026 Jul 3;17:1777211. doi: 10.3389/fpsyg.2026.1777211 (PMC13376299; doi:10.3389/fpsyg.2026.1777211)
Supplement: Supplementary file 1 [file Table_1.docx]

**Table S1**. Construction and Dichotomization Criteria of Network Nodes

| ***Variable*** | ***Source Instrument*** | ***Aggregation Level*** | ***Dichotomization Criterion*** |
| --- | --- | --- | --- |
| B1:Balanced Constitution | CCMQ-30 | Subscale | Raw scores for each constitution dimension were calculated by summing the corresponding item scores. Standardized transformation scores were then computed according to the official scoring procedure:  Transformation Score=(Raw Score−Number of Items) / (Number of Items×4) ×100  For Balanced Constitution, a value of 1 was assigned when the transformed score of the Balanced Constitution dimension was ≥60 and the transformed scores of all other eight constitution dimensions were <30, consistent with the official classification criteria. All other cases were coded as 0. For each biased constitution type, a value of 1 was assigned when the transformed score was ≥30 (including both constitution tendency and constitution presence), whereas scores <30 were coded as 0. |
| B2:Qi-deficiency Constitution |  |  |  |
| B3:Yang-deficiency Constitution |  |  |  |
| B4:Yin-deficiency Constitution |  |  |  |
| B5:Phlegm-dampness Constitution |  |  |  |
| B6:Damp-heat Constitution |  |  |  |
| B7:Blood stasis Constitution |  |  |  |
| B8:Qi stagnation Constitution |  |  |  |
| B9:Inherited Special Constitution |  |  |  |
| P1:Depression | BDI | Total scores | 1: Total score ≥ 10 0: Total score ≦ 9 |
| P2:Anxiety | BAI | Total scores | 1: Total score ≥ 8 0: Total score ≦ 7 |
| P3:Suicidal Ideation | SIOSS | Total scores | 1:​ Total score ≧ 12​ AND​ Concealment score < 4​ (indicating active suicidal ideation). 0:​ Otherwise. |
| P4:Suicidal Behavior | SBQ-R | Total scores | 1:​ Total score ≥ 7 0:​ Total score < 7 |
| P5:Emotional Awareness | DERS | Subscale | Responses were dichotomized at the item level. Scores of 1–2 were coded as 0, indicating low endorsement of emotion regulation difficulties, whereas scores of 3–5 were coded as 1, indicating the presence of the corresponding difficulty. |
| P6:Emotional Clarity |  |  |  |
| P7:Acceptance of Emotional Responses |  |  |  |
| P8:Impulse Control Difficulties |  |  |  |
| P9:Difficulties in Goal-directed Behavior |  |  |  |
| P10:Limited Access to Effective Emotion Regulation Strategies |  |  |  |
| P11:Psychological Resilience | CD-RISC | Total scores | 1: Total score ≦71 0: Total score ≧ 71 |
| P12:Insight Thinking | DIS | Total scores | Responses were dichotomized at the item level prior to construct formation. Scores of 1–3 were coded as 0, indicating a relatively low level of dispositional insight, whereas scores of 4–7 were coded as 1, indicating the presence of the corresponding insight-related characteristic. |
| S1:Childhood Trauma Experiences | CTQ-SF | Total scores | Responses were dichotomized at the item level. Scores of 1 (“Never True”) were coded as 0, indicating the absence of the corresponding adverse childhood experience, whereas scores of 2–5 were coded as 1, indicating the presence of the experience. |
| S2:Perceived Stress | PSS | Total scores | 1: Total score ≥ 29 0: Total score ≦ 28 |
| S3:Family Support | PSSS | Subscale | Responses were dichotomized at the item level. Scores of 1–4 were coded as 0, indicating low perceived social support, whereas scores of 5–7 were coded as 1, indicating the presence of substantial perceived support. |
| S4:Peer Support |  |  |  |
| S5:Other Support |  |  |  |

*Note.* Two types of dichotomization procedures were used. First, for instruments with established clinical, screening, or official classification criteria (e.g., CCMQ-30, BAI, BDI, SIOSS, SBQ-R, CD-RISC and PSS), binary indicators were generated according to the corresponding recommended thresholds. Second, for instruments without recognized construct-level cutoffs (e.g., DERS, DIS, CTQ-SF and PSSS), item responses were first dichotomized to reflect non-endorsement (0) versus endorsement (1) of the relevant characteristic. Binary construct-level indicators were then derived from the aggregated item information. This strategy was adopted to ensure comparability of network nodes within the Ising framework while preserving the substantive meaning of the original constructs. Consistent with previous Ising-network studies, binary coding was intended to represent the absence versus presence of clinically or theoretically meaningful characteristics rather than arbitrary sample-based categorization (van Borkulo et al., 2014).
