# Supplementary material for: Pathways for the precise prevention and improvement of mental health among university freshmen: a network analysis and simulated intervention study based on the biopsychosocial model
Source: Front Psychol. 2026 Jul 3;17:1777211. doi: 10.3389/fpsyg.2026.1777211 (PMC13376299; doi:10.3389/fpsyg.2026.1777211)
Supplement: Supplementary file 2 [file Table_2.docx]

**Table S2**. Descriptive Statistics of Each Scale

| ***Variable*** | ***Source Instrument*** | ***N*** | ***Mean*** | ***SD*** | ***Median*** | ***Min*** | ***Max*** |
| --- | --- | --- | --- | --- | --- | --- | --- |
| B1:Balanced Constitution | CCMQ-30 | 3131 | 15.61 | 3.64 | 16 | 4 | 20 |
| B2:Qi-deficiency Constitution |  | 3131 | 8.31 | 3.65 | 8 | 4 | 20 |
| B3:Yang-deficiency Constitution |  | 3131 | 5.65 | 3.07 | 5 | 3 | 15 |
| B4:Yin-deficiency Constitution |  | 3131 | 7.95 | 3.45 | 8 | 4 | 20 |
| B5:Phlegm-dampness Constitution |  | 3131 | 7.36 | 3.34 | 7 | 4 | 20 |
| B6:Damp-heat Constitution |  | 3131 | 6.23 | 2.76 | 6 | 3 | 15 |
| B7:Blood stasis Constitution |  | 3131 | 8.06 | 3.57 | 8 | 4 | 20 |
| B8:Qi stagnation Constitution |  | 3131 | 7.7 | 3.44 | 7 | 4 | 20 |
| B9:Inherited Special Constitution |  | 3131 | 5.76 | 2.76 | 5 | 3 | 15 |
| P1:Depression | BDI | 3131 | 5.15 | 6.63 | 3 | 0 | 63 |
| P2:Anxiety | BAI | 3131 | 4.25 | 6.57 | 2 | 0 | 48 |
| P3:Suicidal Ideation | SIOSS | 3131 | 4.05 | 4.07 | 3 | 0 | 21 |
| P4:Suicidal Behavior | SBQ-R | 3131 | 4.26 | 2.07 | 3 | 3 | 18 |
| P5:Emotional Awareness | DERS | 3131 | 13.64 | 5.06 | 13 | 6 | 30 |
| P6:Emotional Clarity |  | 3131 | 10.92 | 3.76 | 11 | 5 | 25 |
| P7:Acceptance of Emotional Responses |  | 3131 | 13.25 | 5.72 | 13 | 6 | 30 |
| P8:Impulse Control Difficulties |  | 3131 | 13.65 | 4.81 | 13 | 6 | 30 |
| P9:Difficulties in Goal-directed Behavior |  | 3131 | 14.35 | 4.54 | 15 | 5 | 25 |
| P10:Limited Access to Effective Emotion Regulation Strategies |  | 3131 | 17.1 | 6.45 | 16 | 8 | 40 |
| P11:Psychological Resilience | CD-RISC | 3131 | 64.24 | 20.68 | 63 | 0 | 100 |
| P12:Insight Thinking | DIS | 3131 | 23.82 | 5.79 | 23 | 5 | 35 |
| S1:Childhood Trauma Experiences | CTQ-SF | 3131 | 34.32 | 10.24 | 31 | 25 | 97 |
| S2:Perceived Stress | PSS | 3131 | 37.27 | 8.69 | 39 | 14 | 67 |
| S3:Family Support | PSSS | 3131 | 20.96 | 5.57 | 21 | 4 | 28 |
| S4:Peer Support |  | 3131 | 21.36 | 5.36 | 22 | 4 | 28 |
| S5:Other Support |  | 3131 | 20.22 | 5.51 | 20 | 4 | 28 |
