# Supplementary material for: Pathways for the precise prevention and improvement of mental health among university freshmen: a network analysis and simulated intervention study based on the biopsychosocial model
Source: Front Psychol. 2026 Jul 3;17:1777211. doi: 10.3389/fpsyg.2026.1777211 (PMC13376299; doi:10.3389/fpsyg.2026.1777211)
Supplement: Supplementary file 3 [file Table_3.docx]

**Table S3**. Comparison of Each Scale Score Between Male and Female Students

| ***Variable*** | ***Source Instrument*** | ***Male_Mean_SD*** | ***Female_Mean_SD*** | ***t*** | ***p*** | ***Cohens_d*** |
| --- | --- | --- | --- | --- | --- | --- |
| B1:Balanced Constitution | CCMQ-30 | 16.63 ± 3.44 | 14.93 ± 3.61 | 13.259 | <0.001 | 0.478 |
| B2:Qi-deficiency Constitution |  | 7.48 ± 3.58 | 8.87 ± 3.59 | -10.573 | <0.001 | -0.385 |
| B3:Yang-deficiency Constitution |  | 4.69 ± 2.57 | 6.3 ± 3.2 | -15.552 | <0.001 | -0.543 |
| B4:Yin-deficiency Constitution |  | 7 ± 3.34 | 8.6 ± 3.37 | -13.133 | <0.001 | -0.478 |
| B5:Phlegm-dampness Constitution |  | 6.53 ± 3 | 7.93 ± 3.44 | -12.092 | <0.001 | -0.429 |
| B6:Damp-heat Constitution |  | 5.77 ± 2.78 | 6.55 ± 2.71 | -7.757 | <0.001 | -0.284 |
| B7:Blood stasis Constitution |  | 6.77 ± 3.17 | 8.93 ± 3.56 | -17.783 | <0.001 | -0.634 |
| B8:Qi stagnation Constitution |  | 6.88 ± 3.23 | 8.25 ± 3.46 | -11.347 | <0.001 | -0.408 |
| B9:Inherited Special Constitution |  | 5.01 ± 2.49 | 6.27 ± 2.82 | -13.085 | <0.001 | -0.465 |
| P1:Depression | BDI | 4.89 ± 7.05 | 5.33 ± 6.33 | -1.8 | 0.072 | -0.067 |
| P2:Anxiety | BAI | 3.32 ± 6.11 | 4.87 ± 6.79 | -6.63 | <0.001 | -0.237 |
| P3:Suicidal Ideation | SIOSS | 3.85 ± 4.06 | 4.18 ± 4.07 | -2.214 | 0.027 | -0.081 |
| P4:Suicidal Behavior | SBQ-R | 3.88 ± 1.66 | 4.52 ± 2.28 | -9.049 | <0.001 | -0.311 |
| P5:Emotional Awareness | DERS | 14.37 ± 5.87 | 13.14 ± 4.36 | 6.361 | <0.001 | 0.245 |
| P6:Emotional Clarity |  | 10.64 ± 3.8 | 11.12 ± 3.71 | -3.49 | <0.001 | -0.128 |
| P7:Acceptance of Emotional Responses |  | 12.75 ± 5.82 | 13.59 ± 5.63 | -3.975 | <0.001 | -0.146 |
| P8:Impulse Control Difficulties |  | 13.28 ± 4.73 | 13.89 ± 4.85 | -3.481 | <0.001 | -0.126 |
| P9:Difficulties in Goal-directed Behavior |  | 13.86 ± 4.64 | 14.69 ± 4.43 | -4.985 | <0.001 | -0.183 |
| P10:Limited Access to Effective Emotion Regulation Strategies |  | 16.59 ± 6.51 | 17.45 ± 6.38 | -3.664 | <0.001 | -0.134 |
| P11:Psychological Resilience | CD-RISC | 65.1 ± 22.98 | 63.65 ± 18.96 | 1.854 | 0.064 | 0.070 |
| P12:Insight Thinking | DIS | 24.05 ± 6.64 | 23.67 ± 5.13 | 1.704 | 0.089 | 0.065 |
| S1:Childhood Trauma Experiences | CTQ-SF | 34.51 ± 11 | 34.19 ± 9.7 | 0.839 | 0.401 | 0.031 |
| S2:Perceived Stress | PSS | 36.73 ± 8.69 | 37.64 ± 8.66 | -2.872 | 0.004 | -0.105 |
| S3:Family Support | PSSS | 20.87 ± 5.96 | 21.03 ± 5.28 | -0.774 | 0.439 | -0.029 |
| S4:Peer Support |  | 20.85 ± 5.91 | 21.71 ± 4.92 | -4.239 | <0.001 | -0.160 |
| S5:Other Support |  | 20.04 ± 5.98 | 20.34 ± 5.18 | -1.448 | 0.148 | -0.054 |
